# Differences in verbal memory, visuospatial ability and cognitive inhibition among young women using drospirenone and ethinyl oestradiol oral contraceptives versus naturally cycling controls

**DOI:** 10.1017/neu.2025.10053

**Published:** 2026-01-02

**Authors:** Natalia Lagunas, Laura Sánchez-Giraldo, Daniela Grassi, Teresa Diéguez-Risco, Alejandro de la Torre-Luque

**Affiliations:** 1 Department of Legal Medicine, Psychiatry and Pathology, School of Medicine, Ciudad Universitaria, Complutense University of Madrid, Madrid, Spain; 2 Neuroactive Steroids Lab, Cajal Institute, CSIC, Madrid, Spain; 3 Independent Researcher, Columbia; 4 Department of Anatomy, Histology and Neuroscience, School of Medicine, Autonomous University of Madrid, Madrid, Spain; 5 Centro de Investigación Biomédica en Red de Fragilidad y Envejecimiento Saludable (CIBERFES), Instituto de Salud Carlos III, Madrid, Spain; 6 Departament of Social, Work and Differential Psychology, School of Psychology, Complutense University of Madrid, Madrid, Spain; 7 Centro de Investigación Biomédica en Red en Salud Mental (CIBERSAM), Instituto de Salud Carlos III, Madrid, Spain

**Keywords:** Verbal Memory, spatial ability, executive function, menstrual cycle, combined-oral-contraceptives

## Abstract

**Objective:**

This study aimed to investigate the differences on cognitive performance across four cognitive domains – verbal memory, language fluency, visuospatial ability and cognitive inhibition – between drospirenone and ethinyl oestradiol (DRSP/EE) users and naturally cycling women in the luteal phase (LP). The goal was to determine whether hormonal suppression associated with DRSP/EE use is linked to domain-specific cognitive alterations.

**Methods:**

A total of 48 young adult women were assessed: 23 using DRSP/EE (with pharmacologically suppressed endogenous hormonal levels) and 25 naturally cycling during the LP. Participants completed standardised neuropsychological tasks measuring verbal memory, language fluency, visuospatial ability and cognitive inhibition. Group comparisons analyses were conducted.

**Results:**

Significant group differences were observed in verbal memory, visuospatial ability and cognitive inhibition, while no significant group differences were found in language fluency. Women using DRSP/EE showed significantly lower performance in verbal memory (*U* = 165, *p* = 0.009, *r* = 0.38) and visuospatial ability (*U* = 155, *p* = 0.006, *r* = 0.40) tasks compared to naturally cycling women. In contrast, they demonstrated higher performance in cognitive inhibition, quantified by a significantly higher Stroop interference score (*t*(46) = 2.710, *p* = 0.009, *d* = 0.783).

**Conclusion:**

The present findings suggest that the use of DRSP/EE oral contraceptives is associated with differences across specific cognitive domains compared to naturally cycling women in the LP. The observed pattern – lower performance in hippocampus-related domains (verbal memory and visuospatial ability) paired with higher performance on a frontal-lobe-dependent task (cognitive inhibition) – is consistent with existing evidence suggesting that suppression of endogenous ovarian hormones may differentially influence cognitive functions. These behavioural associations underscore the need for further domain-specific research into the long-term cognitive implications of combined oral contraceptives.

## Graphical abstract

**Figure:**
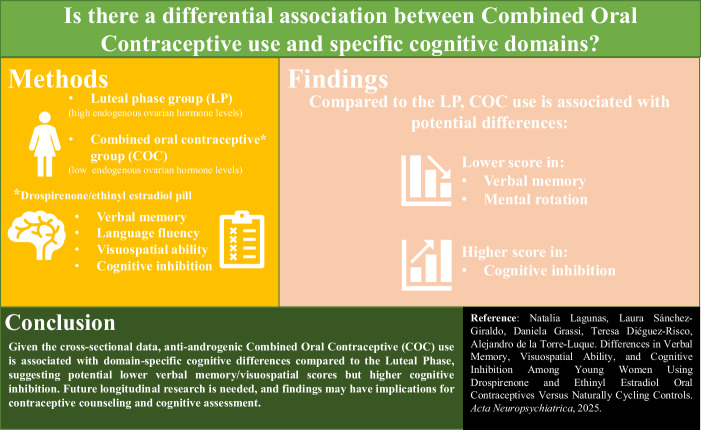

Significant outcomes

- Women using drospirenone and ethinyl oestradiol oral contraceptives were associated with lower performance in verbal memory and visuospatial tasks compared to naturally cycling women.
- Higher performance in cognitive inhibition was observed in anti-androgenic combined oral contraceptive users, suggesting a potential differential association in executive control processes under endogenous hormonal suppression.
- The findings contribute to a growing body of evidence indicating that combined oral contraceptive use may differentially affect specific cognitive domains in women of reproductive age.

Limitations

- The study did not include direct neurobiological measures or hormone level assays, which limits the ability to establish causal neural mechanisms. Similarly, other non-hormonal variables that influence cognitive performance, such as cognitive strategies, mood, intelligence, personality or culture, were not assessed.
- The sample size, while adequate for detecting large effects, may not capture more subtle cognitive differences or interactions.

## Introduction

According to the United Nations, hormonal oral contraception is one of the most widely used contraceptive methods, with approximately 151 million women of reproductive age using it worldwide (United Nations, 2019). Within this category, the combined oral contraceptives (COC) represent a major formulation. COC typically consist of a synthetic progestogen (progestin), such as drospirenone (DRSP), combined with a synthetic oestrogen, specifically ethinylestradiol (EE). They are prescribed not only for birth control but also for managing menstrual or menopausal symptoms and for treating conditions like acne (Davison *et al*., 2013; Brynhildsen, 2014). The contraceptive action of DRSP/EE is achieved by suppressing gonadotropin release from the hypothalamus, thereby inhibiting ovarian stimulation and follicular maturation, and maintaining low levels of endogenous oestradiol and progesterone (Bachmann & Kopacz, 2009; Duffy & Archer, 2018). Synthetic progesterone and synthetic oestrogen exhibit biological effects similar to their natural counterparts by binding to and activating intracellular progesterone and oestrogen receptors. These receptors are widely expressed in key brain regions, including the hippocampus, frontal cortex and hypothalamus, and in major endocrine glands such as the pituitary gland and adrenal glands – areas whose hormonal output further influences brain function (Brinton *et al*., 2008; Weiser *et al*., 2008; Lagunas *et al*., 2022; Pletzer *et al*., 2023). A crucial factor in the brain and cognitive impact of COC stems from the pharmacological diversity among progestins, which differ in their interactions with neuronal receptors and neurotransmitter systems, potentially leading to distinct emotional and cognitive outcomes (Griksiene *et al*., 2022; Pletzer *et al*., 2023; Bencker *et al*., 2025). In that regard, preclinical data in animal models, showed that medroxyprogesterone acetate (MPA), norethindrone acetate (NETA) and segesterone-acetate (SGA) impair spatial learning and memory, while levonorgestrel (LEVO) enhances performance (Braden *et al*., 2017; Bernaud *et al*., 2023). Furthermore, neurogenesis and neuroprotection are increased by LEVO treatment, whereas MPA decreases both (Liu *et al*., 2010; Jayaraman & Pike, 2014). Evidence regarding the cognitive associations of EE alone is limited and inconclusive with outcomes potentially mediated by dose and hormonal status. For instance, high-dose EE treatment has been shown to impair spatial memory in female ovariectomized rats, possibly by reducing basal forebrain cholinergic cells (Mennenga *et al*., 2015). Conversely, high-dose EE increased performance in novel object recognition tasks in intact female rats, and low-dose of EE reduced brain-derived neurotrophic factor mRNA in the hippocampus (Simone *et al*., 2015). There are currently no studies in human females evaluating the isolated cognitive associations of DRSP or EE; their associations have only been measured in COC formulations. Regardless, the overall cognitive associations of COC remain unclear, with studies reporting positive, negative, or neutral outcomes depending on factors such as progestin type, dosage, age and the specific cognitive tasks assessed (Gurvich *et al*., 2023). Specifically in human females, an early study found no differences in domains like memory, attention, visuospatial ability or language between COC users and naturally cycling women assessed in the mid-luteal phase (LP) (Gogos, 2013); but this study did not account for the crucial pharmacological differences among progestins. However, subsequent research has suggested this distinction is critical: when COC users were separated by progestin type, users of androgenic COC (e.g., LEVO) exhibited higher performance in visuospatial ability compared to users of anti-androgenic COC (e.g., DRSP) (Gurvich *et al*., 2020). Preclinical evidence on DRSP further highlights the complexity of the combined formulation. In ovariectomized rats, DRSP administered alone (at low, medium and high doses) increased performance in working memory tasks compared with controls. Furthermore, a moderate, dose-dependent improvement was observed in spatial memory tasks following treatment with medium and high doses (Koebele *et al*., 2022). However, this beneficial effect on spatial memory was reversed when DRSP was co-administered with EE, an outcome potentially linked to the increased expression of glutamate decarboxylase (GAD) observed in the perirhinal cortex following EE administration (Koebele *et al*., 2022).

Given that DRSP/EE is one of the most frequently prescribed progestin combinations in COC (Boehnke *et al*., 2024), and that the current evidence suggests an association between COC use and hippocampus-related tasks contingent on progestin type and dose, a critical knowledge gap remains. To date, few studies have specifically focused on the cognitive associations of DRSP/EE in women of reproductive age, often grouping this formulation with other COC. This study aims to contribute to the existing evidence by evaluating four cognitive domains – verbal memory, language fluency, visuospatial ability and cognitive inhibition – in women using DRSP/EE compared to naturally cycling assessed during the LP, when endogenous ovarian hormone levels are at their natural peak. Verbal memory and visuospatial abilities have been linked to hippocampus function (Bonner-Jackson *et al*., 2015; Wei *et al*., 2016); cognitive inhibition has been associated with the left dorsolateral prefrontal cortex and right cerebellum activity (Okayasu *et al*., 2023); and language fluency with activity in frontal and temporal lobes (Birn *et al*., 2010). Based on preclinical and clinical evidence suggesting a differential association for anti-androgenic progestins and potential negative outcomes for hippocampus-related function, we hypothesised that DRSP/EE use would be associated with significantly lower performance on hippocampus-dependent tasks (verbal memory and visuospatial ability), but would show no significant differences in performance on non-hippocampus-dependent tasks (cognitive inhibition and language fluency) compared to the naturally cycling group.

## Material and methods

### Participants

The study initially evaluated 60 women aged between 18 and 35 years, all fluent in Spanish. All participants met the following general exclusion criteria: a history of neuropsychiatric, neurological or endocrine disorders; sensory impairments; current pregnancy or breastfeeding within the past 12 months and the use of any non-contraceptive medication. Inclusion criteria for the naturally cycling group required regular menstrual cycles (average length of 28–32 days). After applying inclusion and exclusion criteria, the final sample comprised 48 participants: 23 using COC and 25 naturally cycling women. These naturally cycling women were evaluated during LP (menstrual cycle days 18 to 28), which was individually determined based on the forward and backward counts from the start days of both the preceding and subsequent menstrual cycles (Schmalenberger *et al*., 2021). This procedure resulted in the majority of participants being assessed during the mid-LP (*n* = 22, 88%, defined as day −9 to −5 before menstrual onset) and a smaller proportion during the late-LP (*n* = 3, 12%, defined as day -2 to -4 before menstrual onset). Participants in the COC group were assessed during the active pill intake phase (cycle days 5 to 21). COC users were required to have been consistently using a COC containing 3 mg of DRSP and either 20 micrograms of EE (69.6% of the group) or 30 micrograms of EE (30.4% of the group) for a minimum of three consecutive months. The exact duration of treatment was not further quantified. There were no significant differences found between groups regarding key demographic variables: age (COC = 21.7 ± 3.1; LP = 23.2 ± 3.5), years of education (COC = 15.6 ± 1.5; LP = 15.6 ± 1.2) or socioeconomic status (COC = 4 ± 1; LP = 4.2 ± 1.3). A sensitivity power analysis was performed using G*Power (Faul *et al*., 2007) to determine the minimum effect size the study was adequately powered to detect. For a two-tailed independent-samples *t*-test with an α of 0.0125 and a desired power of 0.80, our sample sizes (COC = 23 and LP = 25) were sufficient to detect a minimum effect size of Cohen’s *d* = 0.9, indicating the study was designed to reliably detect effects in the large range (Cohen, 1992).

### Cognitive assessment

Verbal memory: The Hopkins Verbal Learning Test-Revised (HVLT-R) is a validated measure of verbal learning and memory. Participants are read a list of 12 words (4 words from 3 semantic categories) across three acquisition trials. Primary outcome measures include total recall across the three trials, delayed recall (administered 20–25 minutes post-acquisition) and a Recognition Discrimination Index (Arango-Lasprilla *et al*., 2015). This domain is generally recognised as sensitive to gonadal hormone levels (Sultana *et al*., 2025).

Visuospatial ability: Non-verbal reasoning, mental rotation and visuospatial processing were assessed using the Visual Puzzles Subtest of the Wechsler Adult Intelligence Scale-IV (WAIS-IV). This task requires participants to mentally manipulate and select the three pieces (from six response options) that correctly form a given target image; that requires to mentally rotate the candidate pieces to see if they fit and how they fit into the final figure. Scoring is based on both accuracy and time to completion (Wechsler, 2012). Performance on similar visuospatial tasks has been linked to circulating levels of testosterone and oestrogen (Barry *et al*., 2013).

Cognitive inhibition: The Stroop Colour and Word Test (SCWT) assesses processing speed, reading automation, inhibitory control and executive functioning. The SCWT involves three main conditions: (1) Word reading (reading colour names), (2) Colour naming (naming the ink colour), and (3) Interference (naming the ink colour when the word itself names a different colour). The interference coefficient derived from this test is a primary measure of cognitive flexibility and inhibition (Rodríguez Barreto *et al*., 2016). The interference score (calculated from time and errors) has been specifically reported as susceptible to changes related to both exogenous oestrogen administration and endogenous progesterone levels (Krug *et al*., 2006; Hidalgo-Lopez & Pletzer, 2017).

Language fluency: The verbal fluency test requires participants to produce the maximum number of non-proper or non-derived words within a limited time, using either a pre-established letter (phonological fluency) or a specific category (semantic fluency) (Borkowski *et al*., 1967). For the present study, the phoneme ‘S’ and the category ‘fruits’ were administered. The internal reliability for the letter ‘S’ is robust (Cronbach’s *α* = 0.83) (Tombaugh, 1999). Differences in phonological fluency have previously been reported in relation to menstrual cycle phase and COC use (Griksiene and Ruksenas, 2011; Simić & Santini, 2012).

### General procedure

The study was conducted in accordance with the Declaration of Helsinki, and all participants provided written informed consent prior to participation. To ensure consistency in testing conditions, all participants were assessed using a standardised, fixed battery approach (Casaletto & Heaton, 2017), with standardised administration procedures applied across test order, session duration and time of day. Each participant was assessed in a single session, lasting at most 90 minutes, and administered by the same examiner. All assessments were conducted in the afternoon (between 2:00 p.m. and 6:00 p.m.) in the following fixed sequence: (1) HVLT-R Trials 1–3; (2) Visual Puzzles Subtest of WAIS-IV; (3) SCWT; (4) HVLT-R - Delayed Recall and Recognition and (5) VFT. The entire battery was administered using validated, standardised versions.

### Statistics

Shapiro–Wilk’s test was performed to evaluate normality and Levene’s test was used to assess the homogeneity of variances. Group differences were analysed using independent-samples t-tests, for those variables that met the normality principle and for those that did not meet this assumption the non-parametric Mann–Whitney *U* test was used. The magnitude of differences was reported using Cohen’s *d* for the parametric tests (*t*-test), and the non-parametric effect size *r* (calculated as Z/N) for the rank-based tests (Mann–Whitney *U* test). All statistical analyses were performed using SPSS version 30 (IBM Corp., Armonk, NY, USA). Statistical significance was set at *p* < 0.05. Significance levels are reported as follows: *p* < 0.05 (*), *p* < 0.01 (**), and *p* < 0.001 (***). To control for the Family-Wise Error Rate (FWER) due to multiple comparisons across the three dependent variables, the Bonferroni correction was applied. The nominal alpha level was adjusted to *α*_adj_ = 0.0125 (calculated as 0.05/4). *P*-values below this adjusted threshold were considered statistically significant. Data visualisation was performed using OriginPro 2023b (OriginLab Corp., Northampton, MA, USA). Data in figures are presented as mean ± standard error of the mean (SEM) for parametric data, and median and interquartile range (IQR) for non-parametric data.

## Results

### COC use is associated with lower scores in verbal memory and visuospatial ability

**Figure 1.**
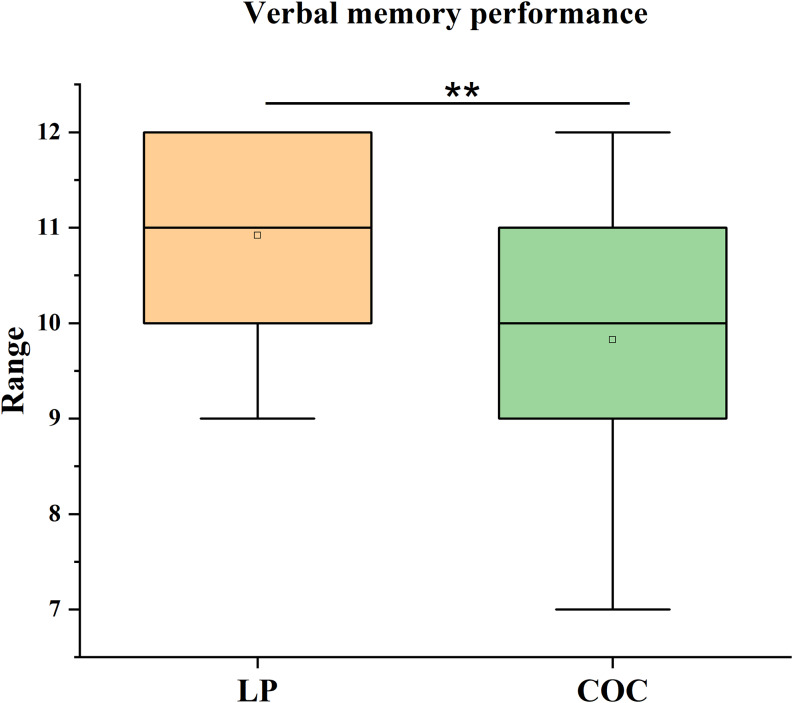
Verbal memory performance. Median scores (IQR) on the recall condition of the Hopkins Verbal Learning Test–Revised (HVLT-R) in the luteal phase (LP) group and the combined oral contraceptive (COC) group. The difference was analysed using the Mann–Whitney *U* test. **p < 0.01, COC vs. LP.

Statistical analysis revealed significant group differences in verbal memory performance, even after applying the Bonferroni correction for multiple comparisons (*α*_adj_ = 0.0125). Specifically, LP group performed significantly higher on the recall condition of the HVLT-R than the COC group (*U* = 165, *p* = 0.009, *r* = 0.38). The effect size was medium. The LP group showed a median score of 11 (IQR = 2), compared to a median score of 10 (IQR = 2) in the COC group (Figure 1).

**Figure 2.**
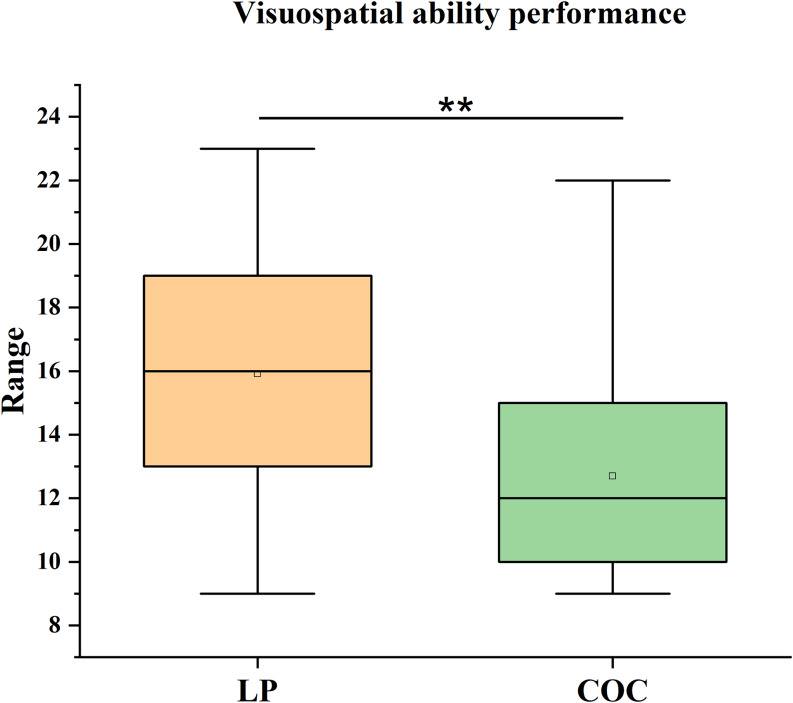
Visuospatial performance. Median scores (IQR) on the visual puzzles subtest of the Wechsler Adult Intelligence Scale – Fourth Edition (WAIS-IV) in the LP and COC groups. The difference was analysed using the Mann–Whitney *U* test. **p < 0.01, COC versus LP.

Similarly, a significant difference was observed in visuospatial ability, as measured by the Visual Puzzles subtest of the WAIS-IV, even after applying the Bonferroni correction for multiple comparisons (α_adj_ = 0.0125). The LP group outperformed the COC group (*U* = 155, *p* = 0.006), with a medium effect size (*r* = 0.40). The LP group showed a median score of 16 (IQR = 7) compared to a median score of 12 (IQR = 5) for the COC group (Figure 2).

### COC use is associated with higher scores in cognitive inhibition

**Figure 3.**
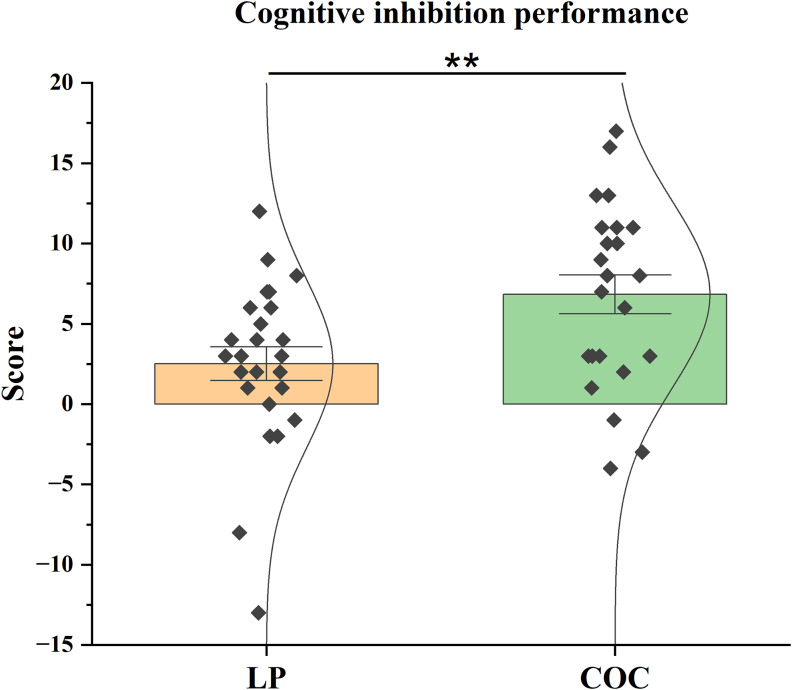
Cognitive inhibition performance. Mean interference coefficient scores (± SEM) on the Stroop Colour and Word Test (SCWT) in the LP and COC groups. The difference was analysed using the *t*-test. **p < 0.01, COC versus LP.

Analysis of cognitive inhibition, assessed via the interference coefficient of the SCWT, showed a significant group difference, even after applying the Bonferroni correction for multiple comparisons (*α*_adj_ = 0.0125). The difference was associated with a medium effect size (*d* = 0.783). The COC group scored significantly higher (*t*(46) = 2.710, *p* = 0.009) than the LP group. The COC group scored *M* = 6.83 (SEM = 1.205), compared to *M* = 2.52 (SEM = 1.046) for the LP group (Figure 3).

**Figure 4.**
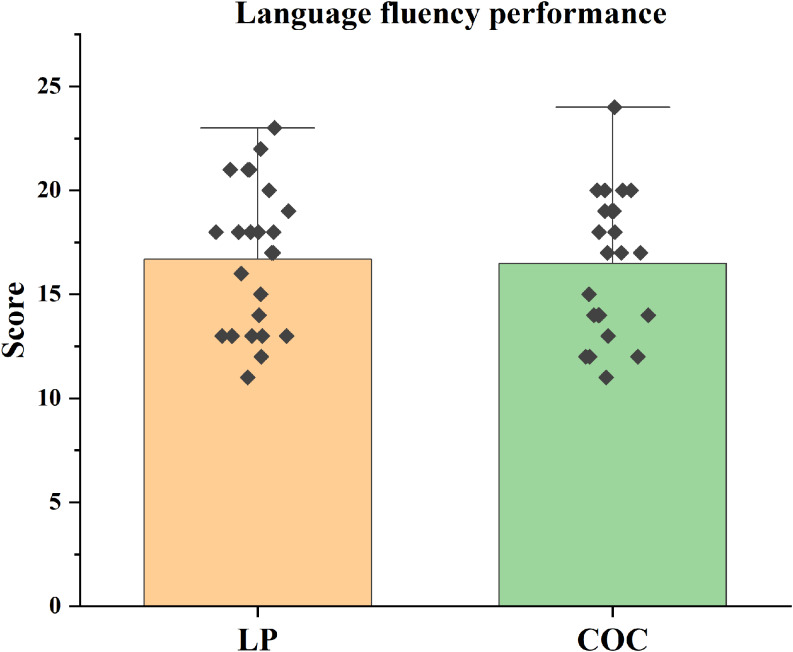
Language fluency performance. Mean language fluency scores (± SEM) on the verbal fluency test (VFT) in the LP and COC groups. The difference was analysed using the *t*-test.

No significant differences were found between groups in language fluency tasks (*p* > 0.0125) (Figure 4).

## Discussion

Our results showed that DRSP/EE use was associated with lower performance in verbal memory and visuospatial ability tasks in comparison to naturally cycling women evaluated in the LP. As indicated in the introduction, the existing literature regarding cognitive associations of COC use is inconclusive; some studies report no differences between COC users and naturally cycling participants, but differences often emerge when COC are separated by progestin action (e.g., androgenic or anti-androgenic) and/or when menstrual cycle phase is assessed (Gurvich *et al*., 2023). For example, a recent study reported no differences in verbal memory performance between COC users and naturally cycling participants (Hochheim *et al*., 2025). However, only 6% of those naturally cycling participants were assessed during the LP, and the COC users were not analysed by the differential androgenic action of progestins.

The findings from the current study suggest that LP is a particularly relevant control phase. Differences in verbal memory performance in naturally cycling women have been linked to differences in left hippocampal activity across the menstrual cycle: increased activity is reported during the LP compared with activity during the follicular phase (Lisofsky *et al*., 2015). Given that the left hippocampus is crucial for verbal memory performance (Bonner-Jackson *et al*., 2015), this is further supported by findings showing a decrease in the magnitude of left hippocampus activity during verbal encoding, which was associated with a decline in endogenous oestradiol levels (Jacobs *et al*., 2016).

Regarding visuospatial abilities, the literature suggests that progestin androgenicity is a key factor in performance. In previous studies, non-COC users outperformed anti-androgenic COC users (Wharton *et al*., 2008; Griksiene *et al*., 2018). Conversely, androgenic COC users reported higher scores than women in the mid-luteal or pre-ovulatory phases (Peragine *et al*., 2020). In contrast, another study reported no differences in visuospatial abilities and verbal memory among COC users (considering androgenicity) and naturally cycling women (Davignon *et al*., 2024). However, in this latter study, naturally cycling women were only assessed during the early follicular and pre-ovulatory phases and not during the LP. Therefore, the menstrual cycle phase of assessment of naturally cycling participants is relevant to explaining our results.

Visuospatial performance has been reported to be sensitive to the hormonal milieu; lower scores were associated with the highest oestrogen levels (whether due to menstrual cycle phase or COC treatment) (Peragine *et al*., 2020). Almost all our participants (88%) were in the mid-LP, when endogenous oestradiol and progesterone levels are expected to be relatively high. In contrast, DRSP/EE pill suppresses ovulation and maintains low levels of these hormones (Bachmann and Kopacz, 2009), but possesses anti-androgenic properties (Edwards & Can, 2024; Sitruk-Ware, 2006). Therefore, in this domain, our results may be better understood in terms of the anti-androgenic action of DRSP. As mentioned previously, androgenic COC users reported higher scores than anti-androgenic COC users.

Moreover, regarding sex differences, it has been profusely documented that males outperformed females in this cognitive domain (Yuan *et al*., 2019; Jacobs *et al*., 2024), and these differences subsequently increase with age (Lauer *et al*., 2019), mirroring the trajectory of androgens levels (Mason *et al*., 2020). Our study did not measure hormones levels, but is well documented that women using anti-androgenic COC have significantly lower circulating androgens and higher sex hormone-binding globulin (SHBG), which decreases bioavailable androgens (Wierman *et al*., 2014; Teal & Edelman, 2021). Therefore, the observed differences in visuospatial performance could be associated with the DRSP/EE anti-androgenic action.

In concordance with this assumption, our findings indicate that COC users demonstrated higher performance in the cognitive inhibition coefficient. As previously described, performance in this task has been associated with activity in the left dorsolateral prefrontal cortex (Okayasu *et al*., 2023). Interestingly, neuroimaging studies report increases in frontal cortex activity associated with anti-androgenic progestins use (Pletzer *et al*., 2023), and previous behavioural studies have reported lower performance in this coefficient during high-hormone menstrual cycle phases (Hatta & Nagaya, 2009). Therefore, the DRSP/EE anti-androgenic action may be linked to increased frontal cortex activity, which could contribute to the observed higher cognitive inhibition performance.

Finally, our results did not indicate differences in verbal fluency. Other studies have reported differences between COC users and non-COC users, but these differences were limited to androgenic COC users (who demonstrated lower scores) in comparison with anti-androgenic COC users and non-users (Griksiene & Ruksenas, 2011). Therefore, our results are in concordance with this previous study.

## Conclusion

Our findings, which must be interpreted with caution given the small sample and the cross-sectional design, suggest that both menstrual cycle phase and anti-androgenic COC use are potentially associated with domain-specific differences in cognition. The LP, naturally characterised by higher endogenous levels of ovarian hormones, was associated with higher scores in verbal memory and visuospatial ability, but conversely, with lower performance in cognitive inhibition. In contrast, the use of anti-androgenic COC, which are known to suppress the endogenous oestrogen, progesterone and androgen levels, was associated with lower scores in verbal memory and visuospatial ability, yet with a higher score in cognitive inhibition. This domain-specific pattern suggests a complex, non-linear interaction between oestrogen, progesterone and androgens and cognitive domains.

While numerous studies have investigated cognitive changes across the menstrual cycle, in relation to various hormonal contraceptives, and compared male and female performance (Le *et al*., 2020; Griksiene *et al*., 2022; Gurvich *et al*., 2023; Pletzer *et al*., 2023), our results specifically highlight the need for more nuanced research. Future studies should employ longitudinal designs to assess causal effects and focus on a wider range of androgenic and anti-androgenic COC formulations to differentiate between the effects of specific progestins. Furthermore, investigation into other contributing variables, such as cognitive strategies, mood, or personality, is essential to increase understanding, as cognitive performance is not a monolithic process.

These findings have real-world relevance for both contraceptive counselling and cognitive assessment. Clinicians should be aware of the potential for minor, domain-specific differences in cognition associated with COC use when counselling patients, particularly those in professions requiring high verbal memory or visuospatial skills. Moreover, cognitive testing batteries should consider menstrual cycle phase or COC status as a variable when establishing baseline cognitive function in reproductive age women.

## Supporting information

Lagunas et al. supplementary material Lagunas et al. supplementary material

## Data Availability

The datasets generated and/or analysed during the current study are available from the corresponding author on reasonable request.

## Supplementary material

The supplementary material for this article can be found here https://doi.org/10.1017/neu.2025.10053.
