# Supplementary material for: Differences in verbal memory, visuospatial ability and cognitive inhibition among young women using drospirenone and ethinyl oestradiol oral contraceptives versus naturally cycling controls
Source: Acta Neuropsychiatr. 2026 Jan 2;38:e9. doi: 10.1017/neu.2025.10053 (PMC13130304; doi:10.1017/neu.2025.10053)
Supplement: Lagunas et al. supplementary material [file S0924270825100537sup001.pdf]

Sociodemographic data (Means, ±SD)

	N	Age	Years of education	Socio-economic status
**COC**	23	21.7 (±3.1)	15,6 (±1.5)	4 (±1)
**LP**	25	23.2 (±3.5)	15.6 (±1.2)	4.2 (±1.3)
**Total**	48	22.5 (±3.3)	15.6 (±1.3)	4.1 (±1.2)

COC treatments

Brand	Users (%)	Drospirenone (mg)	Ethinylestradiol (mg)
Femelle 20 cd	3 (13%)	3	0,02
Yasminiq	7 (30,4%)	3	0,02
Veroniq mini	3 (13%)	3	0,02
Yaxibelle	3 (13%)	3	0,02
Veroniq	1 (4,3%)	3	0,03
Yasmin	2 (8,7%)	3	0,03
Yax	4 (17,4%)	3	0,03
